# Multimodal characterization of male drivers' affect-relevant and workload-related responses in extra-long tunnel driving

**DOI:** 10.3389/fpsyg.2026.1892291

**Published:** 2026-10-07

**Authors:** Han Kang, Shijian He, Wuyang Chen, Zhuming Chen, Kefu Yi, Jie Wang, Chenyu Xu

**Affiliations:** 1 School of Transportation, Changsha University of Science and Technology, Changsha, China; 2 College of Engineering, University of Georgia, Athens, GA, United States; 3 Business School, Soochow University, Suzhou, China

**Keywords:** affective processes, driving workload, multimodal data, naturalistic driving, psychophysiological response, safety risk identification

## Abstract

**Introduction:**

Extra-long tunnels can impose sustained perceptual, physiological, and regulatory demands on drivers, but affect-relevant and workload-related responses are difficult to assess continuously in naturalistic driving because subjective ratings are retrospective and cannot capture fine-grained spatial variation.

**Methods:**

This study developed a multimodal, label-free framework to characterize latent affect-relevant and workload-related response patterns during extra-long tunnel driving. A naturalistic driving experiment was conducted in a 4.9 km tunnel, during which physiological responses, driving behavior, and gaze measures were collected and organized as a third-order tensor (driver × indicator × segment). CANDECOMP/PARAFAC tensor decomposition was then applied to extract the dominant latent structure without subjective labels.

**Results:**

The selected rank-1 solution showed an association with route familiarity. Heart rate and frontal gaze measures contributed most strongly to the latent structure, with additional contributions from vehicle speed and electrodermal activity, suggesting a coupling among autonomic activation, visual attention regulation, and behavioral adaptation. Spatially, elevated factor loadings were observed near tunnel entrances, exits, and several interior segments, indicating that the response patterns observed in male drivers are not limited to transition zones but may also emerge during prolonged tunnel exposure.

**Discussion:**

The study provides exploratory multimodal evidence that may support the interpretation of affect-relevant and workload-related response structures and the identification of spatially differentiated response patterns that may inform further tunnel safety investigation.

## 1 Introduction

Tunnels play an essential role in improving road network connectivity and transportation efficiency, particularly in mountainous areas, urban regions, and complex terrains, where they shorten travel distances, reduce road gradients, and enhance route continuity (Haack, 2002; Zhang et al., 2023). With the rapid expansion of tunnel infrastructure, including the increasing construction of extra-long tunnels, tunnel safety has become a growing concern (International Tunnelling and Underground Space Association (ITA), 2019; Sun et al., 2019). Compared with open roads, tunnels are associated with higher accident risk, more severe congestion, secondary collisions, and delayed emergency responses (Yeung and Wong, 2013; Ma et al., 2009). These risks may become more pronounced as tunnel length increases, since longer enclosed environments can complicate emergency response and impose sustained perceptual and cognitive demands on drivers (Wang et al., 2023; Peña-Garćıa, 2022). Confined spaces, abrupt lighting transitions, and monotonous visual stimuli can increase psychological pressure and emotional tension, leading to attention lapses, delayed reactions, and a higher likelihood of operational errors and accidents (Jiao et al., 2021; Han et al., 2024).

Furthermore, the prolonged and enclosed environment of extra-long tunnels presents more pronounced psychological and physiological challenges for drivers (Yang et al., 2024; Wang et al., 2023). On the one hand, sustained driving over extended periods can impair drivers' perception of external surroundings (Törnros, 1998; Lee et al., 2022; Chatzimichailidou and Dokas, 2016), often disrupting time and spatial awareness and leading to anxiety, tension, and other negative emotional responses (Peña-Garćıa, 2018; Ni et al., 2024). On the other hand, prolonged driving contributes to fatigue accumulation and depletes cognitive resources, which further undermines operational stability and decision-making ability (Liu and Wu, 2009; Ahmad et al., 2023). Moreover, sudden lighting transitions, ambiguous spatial cues, and sound reverberations within extra-long tunnel environments act together to intensify drivers' perceptual and cognitive load (Min and Xu, 2021). In such closed and persistently stressful driving scenarios, drivers must not only cope with external sensory interference but also adapt to a nonlinear workload evolution that unfolds along the tunnel length (Ma et al., 2023b; Qin et al., 2023). This hidden yet progressive accumulation of risk highlights extra-long tunnel driving as a key context for understanding emotion-related, cognitive, and behavioral coupling mechanisms, and underscores the need for detailed modeling of workload dynamics in such environments.

Driving Workload (DWL) is commonly used to characterize the cognitive, psychological, and physiological demands imposed on drivers during task execution, including tension, arousal, and sustained attention under stressful driving conditions. It reflects the driver's information-processing burden and is closely related to operational stability and safety performance (Ma et al., 2023a; Huang et al., 2024). In tunnel environments, previous DWL studies have mainly focused on entrance and exit areas, where abrupt lighting transitions and the black-hole or white-hole effects can produce immediate perceptual stress (Du et al., 2014). However, in extra-long tunnels, workload-related responses may also develop within the tunnel interior due to prolonged exposure, visual monotony, restricted spatial cues, and fatigue accumulation (Farahmand and Boroujerdian, 2018; Dong et al., 2026). Treating the tunnel interior as spatially homogeneous, or representing workload using only a few selected locations, may overlook segment-level variations and potential high-response zones along the driving route. Capturing such spatial variation requires continuous assessment across fine-grained tunnel segments (Xu et al., 2026). However, this remains difficult in naturalistic driving settings. Subjective workload measures, such as NASA-TLX, are usually collected retrospectively and cannot provide reliable segment-level labels throughout the entire tunnel (Karwowski, 2006). As drivers encounter rapidly changing spatial stimuli, it is also impractical to ask them to report workload continuously without interfering with normal driving behavior (Ma et al., 2023a; Mehler et al., 2012). Therefore, this study treats workload as an interpretive construct inferred from convergent evidence across physiological activation, visual attention regulation, and behavioral control, rather than as a directly observed or label-validated workload score.

Given the difficulty of obtaining continuous subjective workload labels, objective workload assessment has increasingly relied on physiological, visual, and driving-behavior signals (Lee et al., 2024). As an implicit psychological construct, DWL cannot be directly observed and must be inferred from measurable responses (Recarte and Nunes, 2003; Tavakoli et al., 2022). Commonly used indicators include heart rate (HR), electrodermal activity (EDA), eye movement measures, and vehicle control features, which reflect physiological arousal, autonomic nervous system activation, visual attention allocation, and operational regulation, respectively (Wei et al., 2023; Deng et al., 2024). Under high-workload or emotionally tense driving conditions, drivers often show increased HR, elevated skin conductance, and altered gaze behavior, indicating higher cognitive demand and sensory-processing load (Mehler et al., 2012; Lohani et al., 2019). However, the use of multimodal indicators also raises a modeling challenge. Many existing studies analyze individual signals separately or combine them through regression, weighted scoring, clustering, or other static approaches (Xing et al., 2021). These methods can provide useful associations, but they often weaken the structural relationships among drivers, indicators, and spatial contexts. For example, correlations between physiological and eye-movement measures may reveal workload-related responses, but they do not explain how these relationships change across different tunnel segments (Reimer et al., 2009). Similarly, manually assigned indicator weights may integrate multiple signals, but they provide limited insight into how each modality contributes to latent workload patterns under different road conditions (Bai et al., 2024). Therefore, examining workload-related responses in extra-long tunnels requires a structured, unsupervised framework that can integrate heterogeneous signals while preserving individual, multimodal, and spatial dimensions of latent response variation.

In addition to challenges related to signal fusion and structural modeling, current studies often overlook individual differences in driving workload responses (Paxion et al., 2014). However, drivers differ significantly in experience level, attentional control, emotional regulation, and physiological sensitivity to stressful driving environments. These factors directly influence how they perceive risk, experience emotional tension and regulate workload under complex driving conditions (Patten et al., 2006). Previous research has shown that experienced drivers are generally more capable of maintaining stable performance under high workload, while novice drivers are more likely to exhibit cognitive delays and behavioral fluctuations (Crundall, 2016). Despite this, most existing models rely on population-averaged strategies. These models fail to account for person-specific variations in physiological responses, behavioral characteristics, and workload evolution patterns. As a result, they may miss high-risk individuals and reduce the effectiveness of personalized detection and intervention (De Waard, 1996). In extra-long tunnels, the driving environment is more prolonged and sensory inputs are more restricted. Under these conditions, individual variability in workload regulation becomes more pronounced. The response patterns of different drivers tend to show stronger individual characteristics (Han et al., 2025). Therefore, modeling workload-related responses can benefit from incorporating individual-level variability and representing the integrated structure of multi-modal indicators and spatial response dynamics. Such a framework supports individual-level characterization of workload-related responses and provides deeper insights into the underlying mechanisms.

To address these challenges, this study formulates extra-long tunnel driving as a label-free, multi-way analysis problem. As illustrated in Figure 1a, naturalistic driving data, including physiological signals, gaze measures, and driving-behavior indicators, were collected during travel through the extra-long tunnel. Rather than treating driving workload as a single aggregated score, we represent tunnel driving as an interaction among three dimensions: individual drivers, multimodal physiological and behavioral indicators, and spatially segmented tunnel sections. This Human-Road-Behavior (HRB) structure provides a unified representation for examining variation in driver-mode loadings, physiological and behavioral responses, and spatial variation along the tunnel. Based on this formulation, as shown in Figure 1b, the collected naturalistic driving data are organized as a third-order tensor with dimensions corresponding to drivers, indicators, and road segments. This representation preserves the relationships among individual heterogeneity, multimodal responses, and spatial driving context. Compared with aggregating multiple indicators into a single workload score or flattening the data into a conventional matrix, the tensor structure retains the original organization of the driving data and allows the three dimensions to be analyzed jointly. In this study, as illustrated in Figure 1c, CANDECOMP/PARAFAC tensor decomposition is applied to the driver × indicator × segment tensor to extract the dominant latent response structure. The decomposed factor vectors provide three interpretable views of this structure: driver-mode loading, indicator contribution, and spatial response distribution. These outputs allow the framework to characterize differences in driver-mode loadings on the shared latent component, identify indicator contributions to that component, and locate tunnel segments with elevated spatial-mode loadings. In this way, the tensor-based analysis connects unlabeled multimodal driving observations with an exploratory characterization of individual differences, signal contributions, and spatial response patterns that may inform further safety investigation. The main contributions of this paper are:

1. A label-free, multi-way analysis framework is established for extra-long tunnel driving. The framework shifts the focus from estimating an overall workload score to characterizing driver-specific loadings on a shared multimodal-spatial response pattern, together with indicator contributions and spatial response distributions relevant to workload and affective processes in transport safety.
2. A multimodal response representation is developed using a driver × indicator × segment tensor. This tensor structure preserves the relationships among individual heterogeneity, physiological-behavioral responses, and spatial driving context under naturalistic tunnel driving conditions.
3. An interpretable tensor-based analysis is conducted to extract latent response structures. The extracted factors are mapped to driver-mode loading, indicator contribution, and spatial distribution, thereby supporting the identification of workload-related driver responses and elevated-response segments in extra-long tunnels.

**Figure 1:**
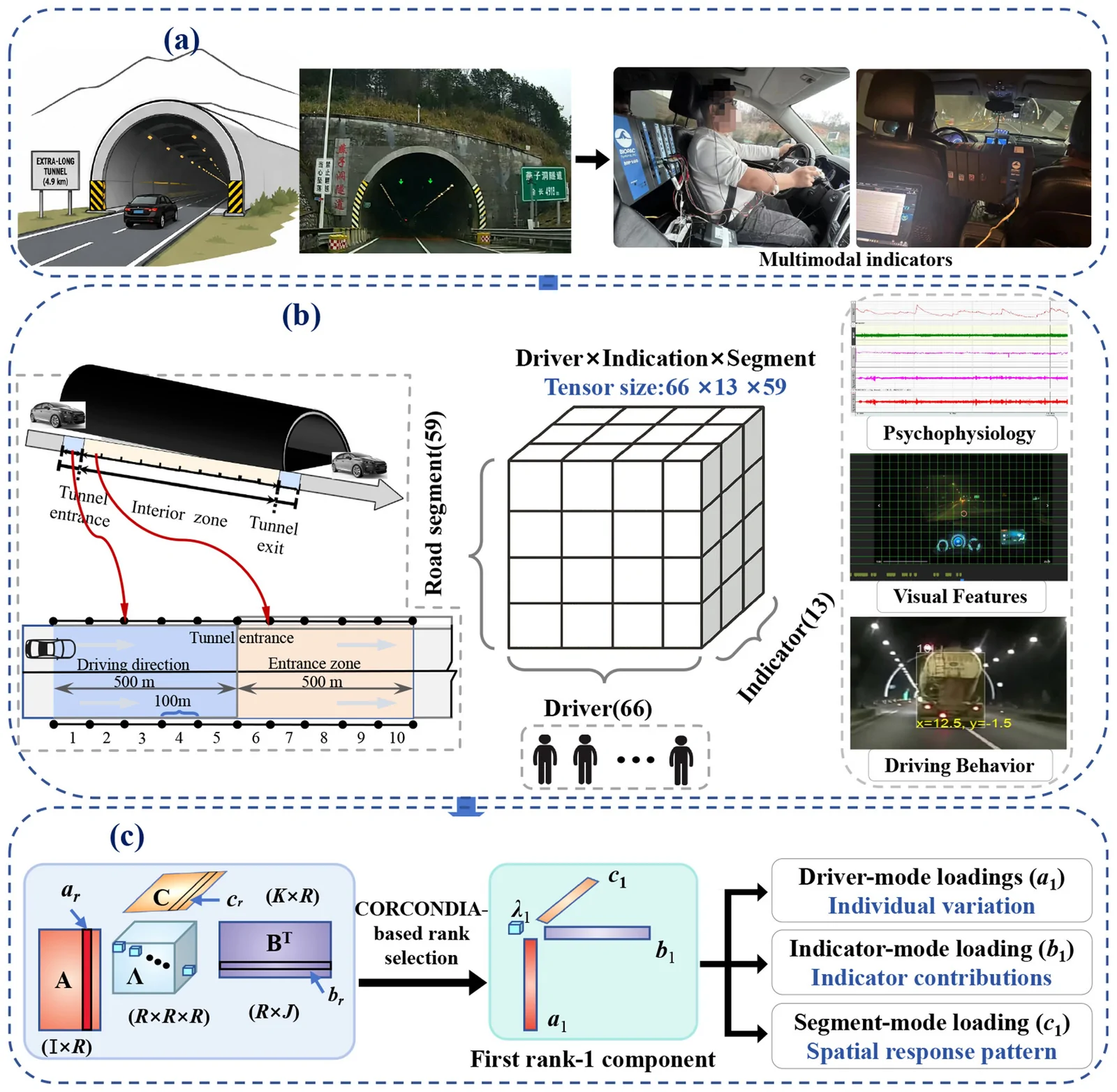
Research workflow of the proposed label-free multimodal analysis framework. **(a)** Naturalistic driving experiment and multimodal data collection, **(b)** third-order tensor construction, and **(c)** CP decomposition and mode-specific factor interpretation.

## 2 Field experiment and data collection

### 2.1 Experimental design

#### 2.1.1 Experimental tunnel and spatial segmentation

This study was conducted in the Yanzidong Tunnel on the Changzhi Expressway in Hunan Province, between the Chenxi and Dajiangkou toll stations. The tunnel is a typical four-lane bidirectional long tunnel with a design speed of 120 km/h, a speed limit of 80 km/h, and a total length of 4.918 kilometers. To capture the spatial variation of workload-related multimodal responses along the entire tunnel, the experimental route was extended to 5.918 kilometers, including a 500-meter approach section before the tunnel, the tunnel main section, and a 500-meter recovery section after the exit. According to the annual average daily traffic and microwave detector data, the selected section operates under Level II or better service conditions, with an average time headway about 3 s. A recognizable leading vehicle was present in most valid HD observations and provided the forward-vehicle reference used to calculate headway distance.

The experimental route was divided into 59 segments (Z1–Z59), generally 100 meter long, with Z54 extending to 118 meter, to support fine-grained spatial representation of workload-related multimodal responses in tensor modeling, as shown in Figure 2. This segmentation offers a trade-off between capturing subtle signal variations and maintaining statistical reliability within each segment.

**Figure 2:**
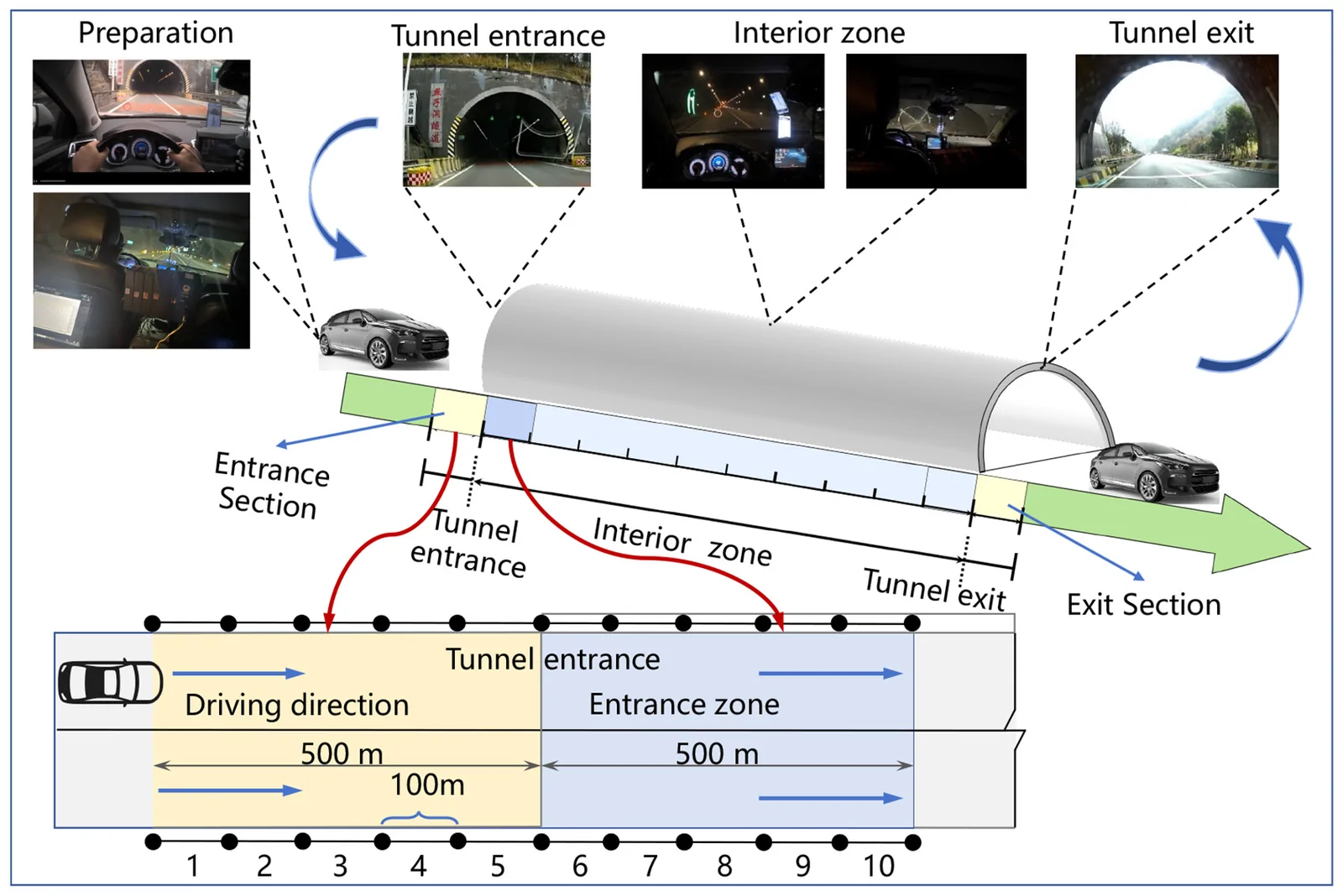
Regional definition of the extra-long tunnel.

#### 2.1.2 Experimental equipment

The experimental setup consisted of a test vehicle, physiological signal acquisition system, eye-tracker, onboard recording devices, laptop, mobile storage devices, and supporting power system, as shown in Figure 3.

**Figure 3:**
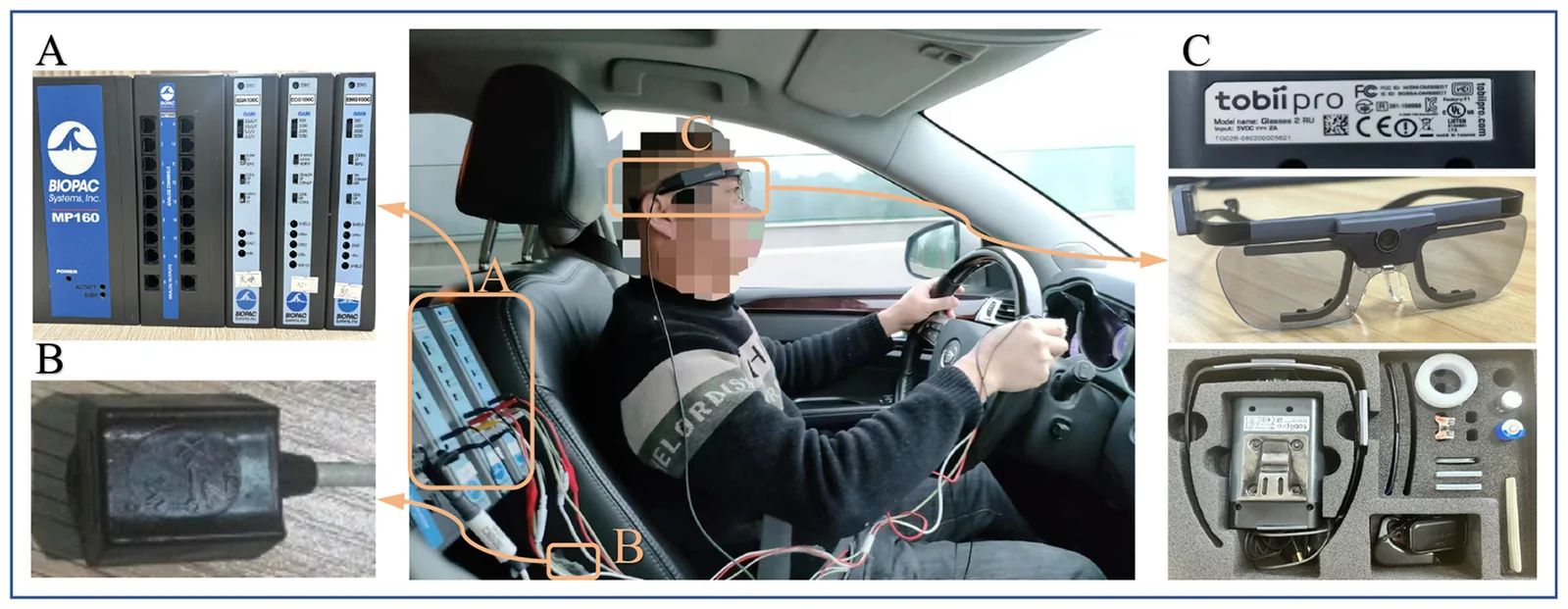
Experiment equipment: **(A)** The Experimental device BIOPAC MP-160, **(B)** Accelerometers, and **(C)** Tobii eye-tracking device. The subject has provided written consent for the publication of this image.

The Cadillac XT5 (2.0T engine, automatic transmission) was selected as the test vehicle. A non-intrusive Tobii eye tracker was employed to capture drivers' visual perception characteristics through scene video coded frame by frame at 24 frames/s, with a vertical field of view of 60° and a horizontal field of view of 80°. The device provided a spatial accuracy of 0.1° and a video resolution of 960 × 720 pixels. A BIOPAC MP160 multi-channel acquisition system recorded ECG, EDA, and triaxial acceleration at 2,000 Hz. All data acquisition processes were centrally controlled by a laptop, and EDA was exported directly using AcqKnowledge (BIOPAC Systems, Inc., Goleta, CA, USA). Additionally, a 360-degree dashboard camera with a frame rate of 60 fps was employed to synchronously record the entire driving process. The video data were used for timestamp alignment and spatial trajectory verification during post-processing.

#### 2.1.3 Participants

To ensure the diversity and representativeness of the sample, a total of 66 drivers were recruited for the on-road study through a combination of direct invitations and public advertisements. All participants were male and held a valid Chinese C1 driver's license, had normal or corrected-to-normal vision, were in good physical condition, and reported no alcohol or medication use that could impair driving ability within 24 h prior to the experiment. Sixty-six experimental data collected in study were valid.

Basic demographic and driving attributes were recorded for each participant, including age (38.1 ± 14.5 years), driving experience (11.2 ± 7.6 years), cumulative driving mileage (297,000 ± 181,000 km), and familiarity with the highway. All attributes were uniformly collected as background variables to support individual-level annotation in subsequent multidimensional data analyses. The sample encompassed drivers across a broad range of ages and experience levels, demonstrating substantial individual variability that is consistent with typical real-world driver populations and providing a solid foundation for constructing the driver mode in the proposed model.

### 2.2 Experimental indicators

DWL is a complex construct arising from the continuous interaction between external traffic demands and internal cognitive, physiological, and perceptual processes (Brookhuis and De Waard, 2010). In constrained environments such as tunnels, visual attention, psychological stress, and motor control are widely recognized as key factors influencing workload (Wang et al., 2024). Accordingly, to select indicators for systematic analysis of workload-related multimodal responses, we referred to existing theoretical insights into the mechanisms of workload formation and considered the perceptual-control characteristics typical of tunnel driving. A set of multimodal indicators with well-established psychological relevance and dynamic sensitivity were selected for analysis, as detailed below. Table 1 provides an overview of the data types and their definitions.

**Table 1:** Classification of driver features and corresponding indicators.

Feature category	Indicator names	Indicator definition	Units
Physiological and psychological response	Heart Rate, HR	Formal-segment mean HR minus the driver's two-run normal-road reference baseline	bpm
	Electrodermal Activity, EDA	Formal-segment mean EDA minus the driver's two-run normal-road reference baseline	μS
Driving Behavior	Vehicle speed, Speed	Segment-level vehicle speed extracted directly from the 360-degree driving recorder	km/h
	Acceleration, Acc	Measured longitudinal component of the BIOPAC MP160 triaxial acceleration	m/s^2^
	Headway Distance, HD	Detected leading-vehicle longitudinal distance calculated from forward video	m
Visual perception	Mean Continuous Gaze Duration—M Region, MAV	Mean duration of continuous gaze episodes on main forward area	ms
	Mean Continuous Gaze Duration—R Region, RAV	Mean duration of continuous gaze episodes on right console	ms
	Mean Continuous Gaze Duration—F Region, FAV	Mean duration of continuous gaze episodes on dashboard	ms
	Mean Continuous Gaze Duration—L Region, LAV	Mean duration of continuous gaze episodes on left mirror	ms
	AOI Gaze-Frame Count—M Region, MA	Cumulative 24-frames/s coded frames assigned to the M AOI within a road segment	frames/ segment
	AOI Gaze-Frame Count—R Region, RA	Cumulative 24-frames/s coded frames assigned to the R AOI within a road segment	frames/ segment
	AOI Gaze-Frame Count—F Region, FA	Cumulative 24-frames/s coded frames assigned to the F AOI within a road segment	frames/ segment
	AOI Gaze-Frame Count—L Region, LA	Cumulative 24-frames/s coded frames assigned to the L AOI within a road segment	frames/ segment

#### 2.2.1 Psychophysiological features

This study employed HR and EDA to capture autonomic nervous system responses triggered by environmental stimuli during driving, serving to characterize physiological responses relevant to workload.

(1) Heart Rate (HR)

Heart rate refers to the number of heartbeats per unit time, typically measured in beats per minute (bpm). During driving, HR is jointly modulated by changes in external traffic conditions and the driver's internal alertness level. As a real-time physiological signal reflecting sympathetic nervous system activation, it is one of the most widely used indicators for assessing variations in driving workload (Arutyunova et al., 2024). HR emphasizes instantaneous levels over shorter time windows, making it more sensitive to physiological arousal and mental tension at the segment level. When entering confined environments such as tunnels, drivers often experience a sharp increase in HR due to abrupt visual transitions, indicating elevated physiological load.

(2) Electrodermal Activity (EDA)

Electrodermal activity refers to changes in skin conductance resulting from sympathetic nervous system activation and sweat gland activity, typically measured in microsiemens (μS) (Critchley, 2002; Boucsein, 2012). EDA is closely associated with emotional arousal, psychological stress, and vigilance, and is widely recognized as a key indicator of mental workload variation. Due to its high sensitivity to environmental stimuli, EDA shows pronounced fluctuations under stressful or anxiety-inducing driving conditions (Yang et al., 2021), making it particularly effective for identifying short-term stress responses triggered by sudden luminance changes or spatial compression in tunnel environments.

#### 2.2.2 Driving behavior features

To characterize drivers' operational responses and behavioral regulation strategies during tunnel driving, this study introduces three dynamic behavioral indicators: vehicle speed, acceleration, and headway distance.

(1) Vehicle Speed (Speed)

Speed is a key parameter reflecting a driver's macro-level control strategy and adaptation to road conditions, typically measured in kilometers per hour (km/h). Variations in speed directly affect perceptual load, decision-making complexity, and operational effort. During tunnel driving, speed regulation is influenced by abrupt changes in illumination, spatial confinement, and psychological stress. Behavioral responses such as deceleration before entry, speed adjustment in the mid-tunnel section, and acceleration after exit may reflect adaptation to changing driving demands (Manser and Hancock, 2007).

(2) Acceleration (Acc)

Acceleration, measured as the longitudinal component of triaxial acceleration acquired by the BIOPAC MP160 at 2,000 Hz, reflects the frequency and magnitude of a driver's longitudinal control adjustments. It serves as a behavioral indicator of the driver's responsiveness to changing traffic environments. Under high workload conditions or increased environmental uncertainty, drivers often exhibit frequent acceleration and deceleration maneuvers to cope with intensified perceptual-motor demands. Such fluctuations not only reduce vehicle stability but also impose a higher operational burden, contributing to elevated overall driving workload (Ariën et al., 2013).

(3) Headway Distance (HD)

Headway distance, measured in meters (m), represents the longitudinal spacing between the ego vehicle and the vehicle ahead, serving as a key behavioral indicator of driver vigilance and risk perception. In tunnel environments, constrained visibility and non-uniform lighting conditions intensify visual and cognitive load, requiring drivers to more frequently assess and regulate their following distance. A shorter headway often reflects a heightened monitoring state and may indicate increased stress or vigilance in maintaining safe spacing under perceived risk (Radhakrishnan et al., 2022).

#### 2.2.3 Visual perception features

Visual perception serves as the primary channel for external information acquisition during driving, accounting for over 70% of incoming traffic-relevant cues (Senders et al., 1967). To analyze drivers' attentional distribution and perception strategies during extra-long tunnel driving, this study extracts several eye-tracking features: gaze area, AOI gaze-frame count and mean continuous gaze duration. These indicators are used to capture spatial variations in visual attention and information processing that may be relevant to workload.

(1) Gaze Area

Gaze area reflects the spatial distribution of a driver's visual attention and serves as a key indicator for assessing environmental awareness and driving strategies. In complex or uncertain road conditions, drivers tend to focus on specific regions to obtain critical information and respond effectively. Previous studies have shown that variations in gaze area are closely related to mental workload and perceptual prioritization. These variations become especially prominent in tunnel environments, where lighting transitions and spatial confinement significantly affect attention allocation (Underwood et al., 2003).

To investigate male drivers' visual attention patterns during tunnel driving, this study divided gaze regions into four categories based on roadway structure and prior research, as shown in Figure 4. These include the main forward area (M region), left mirror (L region), dashboard area (F region), and right console (R region). The division criteria take into account road markings, lighting infrastructure, and the internal layout of the vehicle, ensuring spatial consistency and comparability of gaze data.

**Figure 4:**
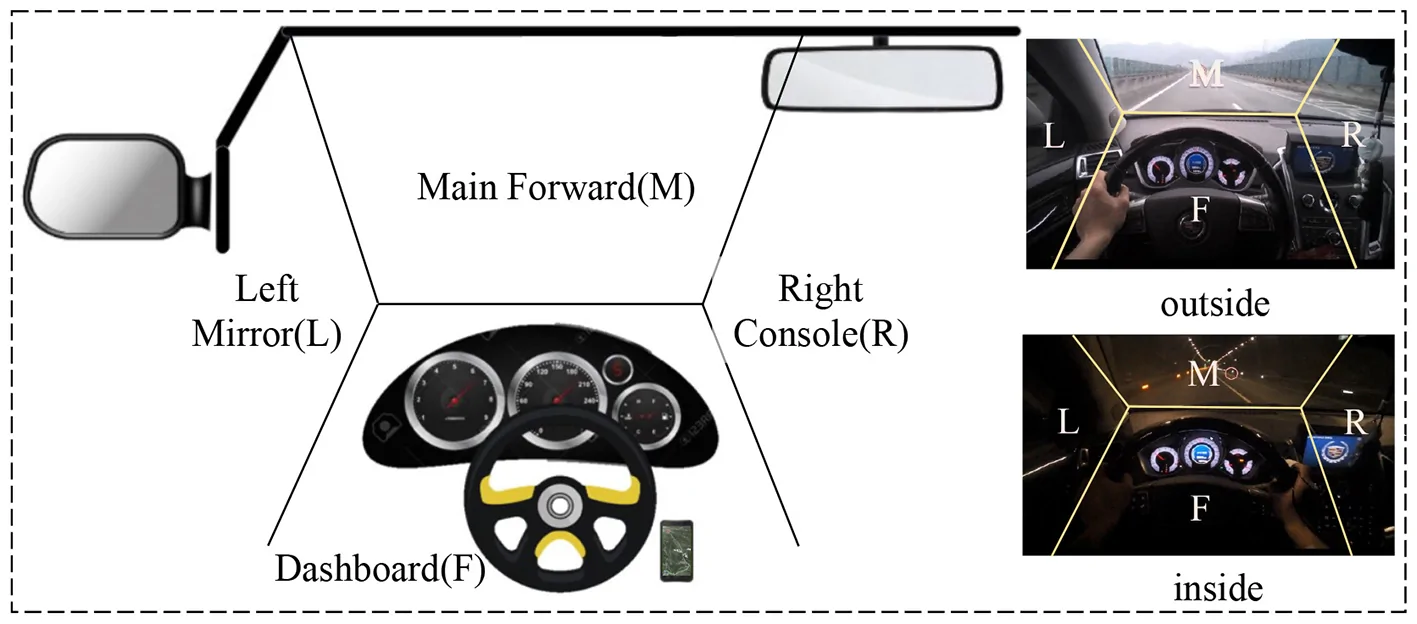
Definition of driver gaze zones.

(2) AOI Gaze-Frame Count

AOI gaze-frame count is the cumulative number of 24-frames/s scene-video frames assigned to an AOI within each predefined road segment and represents segment-based cumulative gaze allocation. Spatial variation in these counts provides a workload-related description of visual allocation across road segments and AOIs.

(3) Mean Continuous Gaze Duration

Mean continuous gaze duration is calculated from maximal consecutive same-AOI frame sequences within each road segment, as defined in Section 2.4, and is measured in milliseconds (ms). Longer continuous gaze durations may reflect greater cognitive-resource allocation for judgment and decision-making and can be workload-related under demanding conditions. In more challenging environments, extended continuous gaze duration is often needed to extract critical information and enhance cue perception.

### 2.3 Experimental procedure

All experiments were conducted during clear daytime conditions to minimize environmental variability. The standardized procedure was as follows:

1. Device initialization: The vehicle was moved to a designated setup area. The windshield was cleaned, and all sensors were installed and tested, including the dashcam, BIOPAC system, and eye-tracker.
2. Subject preparation: Upon arrival, participants were assisted by researchers in wearing the physiological sensors and eye-tracker. A short familiarization drive was conducted to reduce discomfort.
3. Formal test: Prior to entering the tunnel, the participant was briefed on the route and key precautions. All data collection systems were activated simultaneously, and the participant was instructed to drive as usual.
4. Real-time supervision: A researcher remained in the passenger seat to monitor equipment status and ensure the driver did not talk or use distracting electronic devices.
5. Data retrieval: After completing the route, the participant was helped to remove the equipment. Data were backed up immediately to ensure completeness and integrity.

All participants signed written informed consent and received monetary compensation of approximately $30 upon completion. This experiment was approved by the relevant ethics committee, and all procedures followed standard guidelines for human subject research.

### 2.4 Data preprocessing

The driving route comprised 59 predefined spatial segments, generally 100 meter long, with Z54 extending to 118 meter. All missing-, invalid-, and artifact-sample handling was performed at the raw-sample or raw-frame level before segment aggregation. The combined proportion of raw missing and invalid samples was below 1%. For each driver-trial HR/EDA series, the 3σ Pauta rule flagged samples satisfying Equation 1:


$$\begin{array}{l} |x-\mu |>3\sigma , \end{array}$$ (1)

where μ and σ are the mean and standard deviation of the corresponding series. A flagged value was classified as an acquisition artifact when it coincided with a documented signal interruption or appeared as an isolated exceedance with adjacent valid samples within the 3σ limits. Temporally continuous deviations were retained for segment aggregation. Missing and invalid samples in continuous signals, including acquisition artifacts identified by the preceding rule, were reconstructed by cubic Hermite interpolation before segment aggregation.

Gaze-point calibration and equipment function checks were completed before data collection. Missing and invalid eye-tracking frames were handled at the raw-frame level before aggregation. The eye-tracking scene video was coded frame by frame at 24 frames/s into four areas of interest (AOIs): M, L, R, and F. Let $F_{ik}$ denote the set of coded frames for driver *i* in segment *k* after raw-frame processing, and let *A*_*if*_ denote the AOI label assigned to frame *f*. The cumulative AOI gaze-frame count was calculated as shown in Equation 2:


$$\begin{array}{l} C_{iak}=\underset{f\in F_{ik}}{\sum }\text{1}\left(A_{if}=a\right),\;a\in \left\{M,L,R,F\right\}. \end{array}$$ (2)

Accordingly, *C*_*iMk*_, *C*_*iRk*_, *C*_*iFk*_, and *C*_*iLk*_ correspond to MA, RA, FA, and LA, respectively, with units of frames/segment. With the predefined road segment serving as the analysis unit, *C*_*iak*_ is the pure sum of frames assigned to AOI *a* within segment *k*. Speed entered the tensor as a separate indicator, allowing the model to jointly represent segment-level cumulative gaze allocation and vehicle-speed variation.

A maximal consecutive sequence of frames assigned to the same AOI within a segment defined one continuous gaze episode. If episode *e* for driver *i*, AOI *a*, and segment *k* contained *n*_*iake*_ frames, its duration was as shown in Equation 3:


$$\begin{array}{l} t_{iake}=\frac{1000}{24}n_{iake}\text{ms}. \end{array}$$ (3)

*E*_*iak*_ denotes the number of continuous gaze episodes for driver *i*, AOI *a*, and segment *k*. The mean continuous gaze duration was calculated as shown in Equation 4:


$$D_{iak}=\{\begin{array}{ll} \frac{1}{E_{iak}}\sum_{e=1}^{E_{iak}}t_{iake}=\frac{1000}{24E_{iak}}\sum_{e=1}^{E_{iak}}n_{iake}, & E_{iak}\geq 1,\\ {}[6pt]0, & E_{iak}=0. \end{array}$$ (4)

A value of zero was assigned when no continuous gaze episode occurred in AOI *a* within segment *k*. *D*_*iMk*_, *D*_*iRk*_, *D*_*iFk*_, and *D*_*iLk*_ correspond to MAV, RAV, FAV, and LAV, respectively. Every continuous frame sequence contributed to the calculation, with a temporal resolution of 1, 000/24 ≈ 41.7 ms.

Speed was extracted directly from the 360-degree driving recorder. A BIOPAC MP160 recorded triaxial acceleration at 2,000 Hz, and the longitudinal component was used as Acc. Headway distance (HD) was calculated from the recorder's forward video using an author-developed program as the longitudinal distance to the detected leading vehicle. Most valid observations contained a recognizable leading vehicle. Frames lacking an HD estimate entered the raw-level missing-data processing described above.

ECG and EDA were acquired using a BIOPAC MP160 at 2,000 Hz. ECG was amplified 1,000 times, and HR was calculated as shown in Equation 5:


$$\begin{array}{l} HR=\frac{60}{RR}, \end{array}$$ (5)

where *RR* is the R–R interval in seconds. EDA was exported directly using AcqKnowledge. Each driver completed two 500-meter normal straight-road reference runs. After the same quality-control procedure, *b*_*ijr*_ denotes the valid-sample mean for driver *i*, physiological indicator *j*, and reference run *r*. The individual physiological baseline was shown in Equation 6:


$$\begin{array}{l} B_{ij}=\frac{1}{2}\overset{2}{\underset{r=1}{\sum }}b_{ijr},\;j\in P,\;P=\left\{HR,EDA\right\}. \end{array}$$ (6)

For each formal road segment, the baseline-referenced physiological value was shown in Equation 7:


$$\begin{array}{l} d_{ijk}={\bar{x}}_{ijk}-B_{ij},\;j\in P. \end{array}$$ (7)

Here, ${\bar{x}}_{ijk}$ is the processed formal-segment mean for driver *i*, physiological indicator *j*, and road segment *k*; *r* indexes the two reference runs. HR and EDA entered the tensor through *d*_*ijk*_.

Let *q*_*ijk*_ denote the final processed segment-level value for driver *i*, indicator *j*, and segment *k*. For HR and EDA, *q*_*ijk*_ = *d*_*ijk*_; for the remaining indicators, *q*_*ijk*_ is the corresponding segment mean for continuous measures, cumulative AOI gaze-frame count, or AOI mean continuous gaze duration. Each of the 13 indicators was globally standardized across all drivers and all road segments to establish a common numerical scale as shown in Equation 8:


$$\begin{array}{l} {\tilde{x}}_{ijk}=\frac{q_{ijk}-\mu_{j}^{\left(q\right)}}{\sigma_{j}^{\left(q\right)}}, \end{array}$$ (8)

where $\mu_{j}^{\left(q\right)}$ and $\sigma_{j}^{\left(q\right)}$ are the mean and standard deviation of indicator *j* across all drivers and road segments. The resulting standardized tensor was used for CP decomposition.

## 3 Methodology

In transportation behavior research, the increasing use of multimodal data has highlighted the effectiveness of structured modeling techniques in capturing complex interactions across dimensions. Unlike conventional aggregation or matrix-based modeling, the tensor-based approach preserves the original multi-way organization of the data rather than reducing the driver, indicator, and spatial dimensions to a single score or a two-dimensional representation. This enables inter-individual variation, multimodal indicator contributions, and segment-level response patterns to be examined jointly in a label-free setting.

To represent the relationships among driver identity, behavioral indicators, and spatial segments, this study adopts a HRB interaction framework. The overall behavioral representation is formulated as a multivariate function in Equation 9:


$$\begin{array}{l} L=f\left(x_{1},x_{2},\ldots ,x_{n},t,s\right) \end{array}$$ (9)

where *x*_1_~*x*_*n*_ are multimodal indicators, *t* denotes the driver, and *s* represents the spatial segment.

This function *f*(·) entails complex, high-dimensional couplings and nonlinear characteristics that are difficult to capture using conventional linear approaches. To this end, a third-order tensor structure is constructed as a driver × indicator × segment tensor, forming the basis for subsequent unsupervised factor analysis.

### 3.1 Third-order tensor construction

To systematically capture the interactions among individual differences, multimodal responses, and spatial variations in the tunnel driving process, we construct a third-order tensor $X\in {\mathbb{R}}^{I\times J\times K}$ to organize the collected experimental data into a unified structure, as shown in Figure 5. The tensor is defined as in Equation 10:


$$\begin{array}{l} X=\left[x_{ijk}\right]\in {\mathbb{R}}^{I\times J\times K} \end{array}$$ (10)

**Figure 5:**
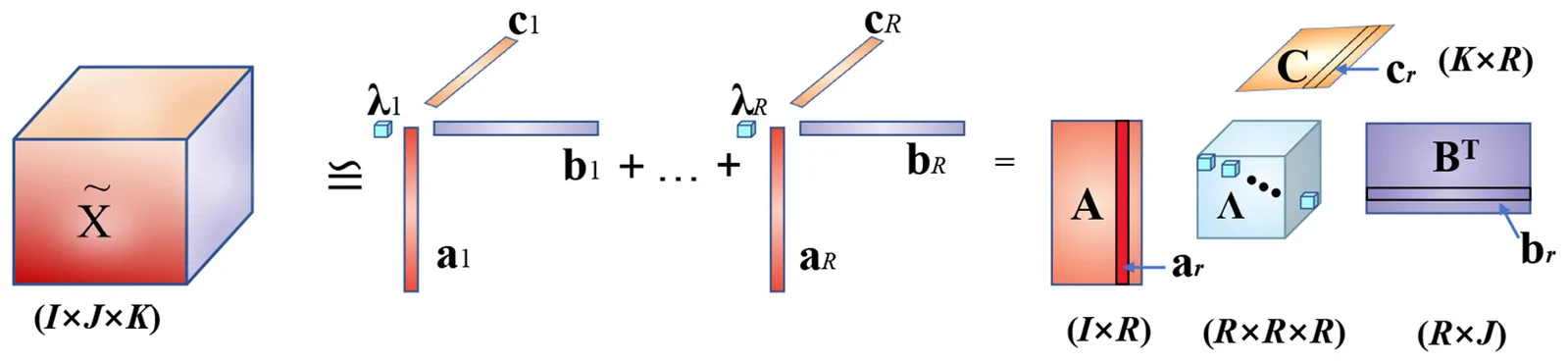
CP tensor decomposition.

where:

- *I*: Driver dimension, representing individual participants (*i* = 1, …, *I*);
- *J*: Indicator dimension, covering physiological signals, driving behavior, and visual perception measures (*j* = 1, …, *J*);
- *K*: Spatial segment dimension, dividing the full tunnel into 59 predefined segments, generally 100 meter long, with Z54 extending to 118 meter (*k* = 1, …, *K*).

The tensor element *x*_*ijk*_ represents the modality-specific processed segment-level value of the *j*-th indicator for the *i*-th driver in the *k*-th segment. Global *Z*-score standardization was applied as defined in Section 2.4 and Equation 8. The resulting standardized tensor $\tilde{X}$ preserves the semantic dependencies across the three modes of driver, indicator, and segment, forming the basis for the subsequent unsupervised modeling process.

### 3.2 CP tensor decomposition modeling

To extract latent structures from the third-order tensor, we apply Canonical Polyadic (CP) decomposition. This method approximates the standardized tensor $\tilde{X}$ as a weighted sum of *R* rank-one tensors, preserving the three-way data structure. The decomposition is expressed as in Equation 11:


$$\begin{array}{l} \tilde{X}\approx \overset{R}{\underset{r=1}{\sum }}\lambda_{r}{\text{a}}_{r}༚{\text{b}}_{r}༚{\text{c}}_{r} \end{array}$$ (11)

where:

- *R* is the rank of the decomposition, representing the number of latent components;
- λ_*r*_ denotes the weight of the *r*-th component;
- ${\text{a}}_{r}\in {\mathbb{R}}^{I}$, ${\text{b}}_{r}\in {\mathbb{R}}^{J}$, and ${\text{c}}_{r}\in {\mathbb{R}}^{K}$ are the factor vectors along the driver, indicator, and segment dimensions, respectively;
- ༚ denotes the outer product operation.

CP decomposition captures multi-way patterns by preserving the interactions across all three dimensions. Each rank-one component reflects a distinct triadic interaction pattern, enabling low-rank approximation while maintaining structural integrity. As an unsupervised technique, it supports the extraction of latent patterns from high-dimensional multimodal data without requiring labeled supervision. Compared to matrix-based approaches, CP decomposition better represents the structural complexity of multidimensional datasets.

### 3.3 Parameter estimation in CP decomposition

In this study, to estimate the parameters of the CP decomposition model, we adopt the Alternating Least Squares (ALS) algorithm, a widely used iterative method that minimizes the Frobenius norm between the original tensor and its low-rank approximation. The optimization is solved by iteratively updating one factor matrix while keeping the others fixed. Each subproblem reduces to a least-squares formulation. The update procedure includes the following steps shown in Equations 12–14:

1. Fix **B** and **C**, update **A**:

$$\begin{array}{l} \underset{\text{A}}{\min}||{\tilde{X}}_{\left(1\right)}-\text{A}{\left(\text{C}⊙\text{B}\right)}^{⊤}|{|}_{F}^{2} \end{array}$$ (12)

2. Fix **A** and **C**, update **B**:

$$\begin{array}{l} \underset{\text{B}}{\min}||{\tilde{X}}_{\left(2\right)}-\text{B}{\left(\text{C}⊙\text{A}\right)}^{⊤}|{|}_{F}^{2} \end{array}$$ (13)

3. Fix **A** and **B**, update **C**:

$$\begin{array}{l} \underset{\text{C}}{\min}||{\tilde{X}}_{\left(3\right)}-\text{C}{\left(\text{B}⊙\text{A}\right)}^{⊤}|{|}_{F}^{2} \end{array}$$ (14)

Here, ${\tilde{X}}_{\left(n\right)}$ denotes the mode-*n* unfolding of the tensor $\tilde{X}$, and ⊙ represents the Khatri-Rao product. This operation ensures the alignment of latent structure across modes during updates.

The iterative procedure continues until the relative change in reconstruction error satisfies Equation 15:


$$\begin{array}{l} \frac{||X-\hat{X}\left(t\right)|{|}_{F}-||X-\hat{X}\left(t-1\right)|{|}_{F}}{||X|{|}_{F}}<\epsilon , \end{array}$$ (15)

or the maximum iteration number is reached. Here, $\hat{X}\left(t\right)$ is the reconstructed tensor at iteration *t*, and ϵ is a pre-defined threshold. In this study, the ALS optimization was performed with 50 random initializations, a maximum of 2,000 iterations, and a convergence threshold of ϵ = 10^−8^. The reconstruction error was monitored across iterations to evaluate the convergence behavior.

### 3.4 Factor interpretation and multi-dimensional representation

In tensor-based modeling, interpretability of factor structures serves as a crucial bridge between data and mechanism. To reveal the structure embedded in the factorized tensor, we interpret the learned factor matrices **A**∈ℝ^*I*×*R*^, **B**∈ℝ^*J*×*R*^, and **C**∈ℝ^*K*×*R*^, which correspond to the three modes: driver samples, observed indicators, and spatial segments, respectively. Each column of these matrices corresponds to one rank-1 component (latent factor), and the vector entries indicate the mode-specific loading strength on that factor.

1. **Factor Matrix A: Driver-Mode Representation**

Each column vector ${\text{a}}_{r}={\left[a_{1r},a_{2r},\ldots ,a_{Ir}\right]}^{⊤}$ reflects the activation pattern of the *r*-th latent factor across all drivers. The entry *a*_*ir*_ indicates how strongly driver *i* is associated with component *r*, serving as a basis for capturing inter-individual variability. The driver loading is defined in Equation 16:


$$\begin{array}{l} {\text{DriverLoading}}_{ir}=a_{ir},\;\forall i\in \left\{1,2,\ldots ,I\right\},\;r\in \left\{1,2,\ldots ,R\right\} \end{array}$$ (16)

2. **Factor Matrix B: Indicator-Mode Representation**

The matrix **B** encodes the contribution of each indicator to the latent factors. Each vector ${\text{b}}_{r}={\left[b_{1r},\ldots ,b_{Jr}\right]}^{⊤}$ reveals the relative importance of the *J* indicators for explaining component *r*. The indicator contribution is defined in Equation 17:


$$\begin{array}{l} {\text{Contribution}}_{jr}=b_{jr},\;\forall j\in \left\{1,2,\ldots ,J\right\},\;r\in \left\{1,2,\ldots ,R\right\} \end{array}$$ (17)

3. **Factor Matrix C: Spatial Segment-Mode Representation**

The third factor matrix **C** reflects the variation of each component across the *K* tunnel segments. Each ${\text{c}}_{r}={\left[c_{1r},\ldots ,c_{Kr}\right]}^{⊤}$ captures the spatial distribution pattern of the *r*-th latent factor. The segment-level loading is defined in Equation 18:


$$\begin{array}{l} {\text{Spatial\_Load}}_{kr}=c_{kr},\;\forall k\in \left\{1,2,\ldots ,K\right\},\;r\in \left\{1,2,\ldots ,R\right\} \end{array}$$ (18)

By examining these three sets of factor vectors, we construct an interpretable multi-view description of latent patterns, encompassing inter-individual heterogeneity, indicator-wise discriminability, and spatial evolution trends. These factor structures form the foundation for downstream analysis of driver response.

For the selected rank-1 solution, the indicator-mode profile **b**_1_ and segment-mode profile **c**_1_ are shared across drivers, whereas individual variation is represented through the driver-mode loading *a*_*i*1_. Differences in *a*_*i*1_ therefore reflect variation in loading magnitude and, under a fixed sign convention, relative loading direction on the shared component, rather than independently estimated indicator combinations or spatial response shapes for individual drivers.

## 4 Results

This section builds upon the tensor decomposition methodology introduced in Section 3.3, specifically focusing on the structural characteristics of the latent components. To evaluate the reliability and interpretability of different decomposition configurations, the Core Consistency Diagnostic (Corcondia) was employed, which is a widely used criterion for validating CP decomposition models (Bro and Kiers, 2003; Bro, 1997). Corcondia evaluates how closely the estimated core tensor resembles a superdiagonal form, where the most significant values are concentrated along the main diagonal and interference between components is minimal. A Corcondia value approaching 100% is generally interpreted as strong evidence that the decomposition yields a stable and interpretable latent component. Such a result suggests minimal distortion from unintended interactions between tensor modes, which refer to artificial dependencies that may arise when an overly high CP rank causes the decomposition to fit noise or incidental correlations rather than meaningful latent structure. In contrast, lower Corcondia values suggest potential structural misfit, reflecting either an overestimation of the model rank or the presence of latent noise.

Based on this evaluation, candidate CP models with ranks from 1 to 3 were compared using Core Consistency Diagnostic and explained variance. The rank-1 model achieved a CORCONDIA score of 100% and explained 90.54% of the total variation. Rank-2 and rank-3 models increased the explained variance to 93.06% and 94.05%, respectively, but their CORCONDIA scores decreased to 39.51% and –270.79%. Thus, the additional components provided limited gains in model fit while reducing structural consistency. The rank-1 solution was therefore retained as a well-supported and structurally coherent representation of the dominant shared multimodal pattern in the present dataset. The remainder of this chapter presents a detailed examination of its first rank-one component from three perspectives: individual driver responses, multimodal indicator contributions, and spatial variation across tunnel segments.

### 4.1 Driver-mode loading analysis

To reveal the structural characteristics of the primary latent component along the driver mode, this section focuses on the rank-1 component (**a**_1_ vector) derived from the CP decomposition. This vector represents the dominant pattern within the driver dimension of the tensor structure and reflects the primary inter-individual differences. To provide a more intuitive illustration of this structure, Table 2 lists the **a**_1_ scores of all drivers, reflecting individual differences in loading magnitude and relative direction on the shared latent component.

**Table 2:** Driver factor scores on the rank-1 component (**a**_1_ vector).

Sample	Loading	Sample	Loading	Sample	Loading
1	–0.03489	23	0.00720	45	–0.02487
2	–0.03378	24	–0.00008	46	0.02977
3	–0.02614	25	–0.01091	47	0.02066
4	–0.00184	26	–0.00207	48	0.03029
5	–0.00248	27	0.01254	49	–0.00922
6	–0.00823	28	0.00480	50	–0.00713
7	–0.00178	29	0.00530	51	–0.02797
8	–0.00658	30	–0.01711	52	–0.00870
9	–0.01837	31	–0.01296	53	0.00976
10	–0.02960	32	–0.02006	54	0.01050
11	–0.04137	33	0.02735	55	0.00417
12	–0.00773	34	0.01646	56	0.00904
13	–0.01761	35	0.03177	57	0.01502
14	–0.00496	36	0.03684	58	0.01607
15	–0.01555	37	0.03032	59	0.01742
16	–0.01692	38	0.02775	60	0.01270
17	0.00154	39	0.01453	61	–0.00765
18	–0.00023	40	0.02729	62	–0.00195
19	–0.01042	41	0.02813	63	0.00104
20	0.00714	42	0.02743	64	0.00588
21	–0.00733	43	–0.01985	65	–0.00103
22	0.00370	44	–0.02269	66	0.00589

To systematically examine the structure within the driver dimension, Figure 6 illustrates the distribution of **a**_1_ loadings across different driver attributes, including total mileage, driver age, driving experience, and route familiarity. The first three attributes were split based on sample medians, while route familiarity was recorded as a background attribute during data collection. To further examine the relationships between the driver-mode pattern and background attributes, exploratory two-sided Mann–Whitney *U* tests were conducted for the four two-level attribute comparisons, and the resulting *p*-values were adjusted using the Holm procedure. No statistically significant group differences were observed for total mileage, driver age, or driving experience (all *p*≥0.05), whereas route familiarity was significantly associated with the *a*_1_ loadings (*p* < 0.001). Specifically, under the displayed sign convention, drivers unfamiliar with the route showed higher and positively skewed **a**_1_ values, while familiar drivers showed more centered distributions with a tendency toward negative values. Because route familiarity was not used as a label in CP decomposition, this finding is interpreted as an emergent driver-level association that helps explain individual differences reflected by the latent component.

**Figure 6:**
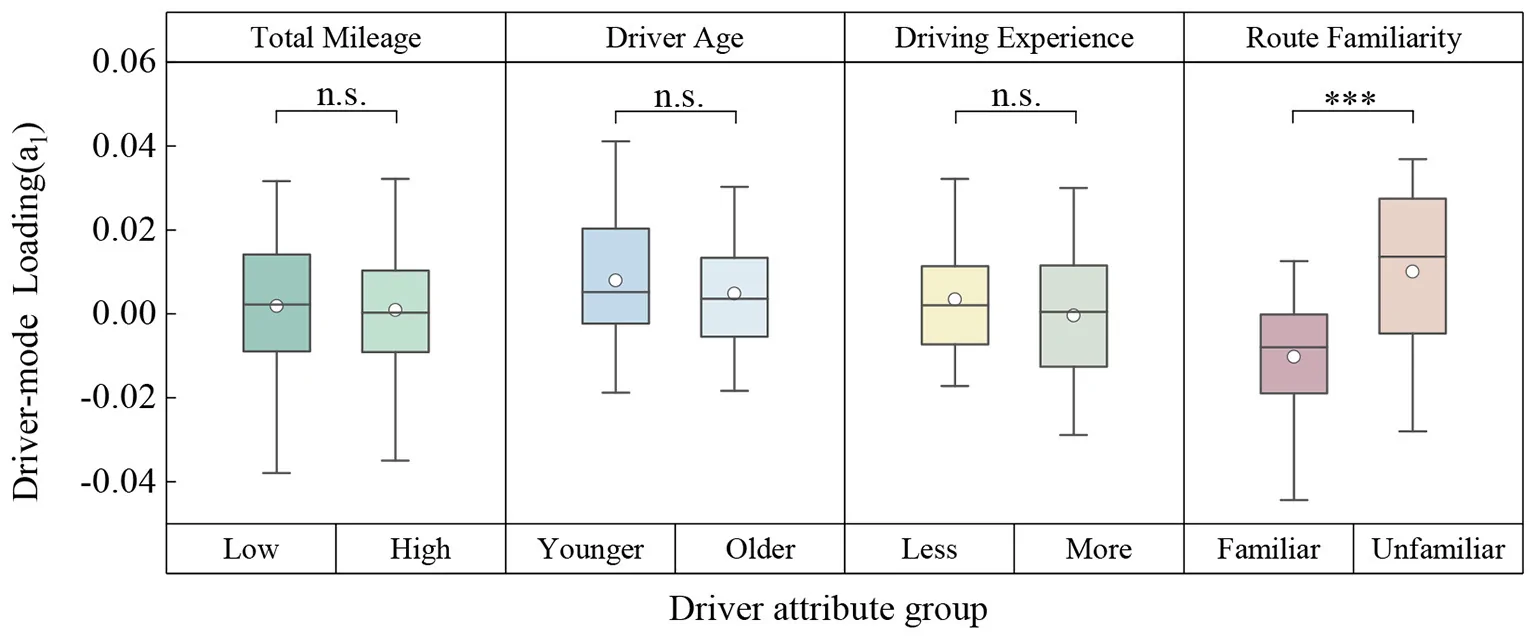
Distribution of driver-mode loadings (**a**_1_) across driver attribute groups. Exploratory comparisons; *p*-values were Holm-adjusted. n.s., *p* ≥ 0.05; ***p < 0.001.

Further insights are provided in Figure 7, which displays the complete distribution of the first latent factor across the driver dimension, illustrating the route-familiarity-related pattern at the individual-driver level and the within-group variability of the **a**_1_ loadings. Each bar represents the component score of an individual driver. For visualization purposes, driver IDs were reordered according to route familiarity so that drivers with the same familiarity level appear consecutively. The distribution reveals a clear bimodal structure, where familiar drivers (IDs 1–32) primarily exhibit negative values with a concentrated spread, while unfamiliar drivers (IDs 33–66) mostly show positive values, with several individuals deviating substantially. Despite the absence of any group labels in model training, the factor scores demonstrate clear group-level separability, reflecting differences in individual driver loadings on the shared component. Additionally, within-group variation is notable. Several drivers in the familiar group show substantially lower scores than the group mean, indicating within-group variation in driver loadings among route-familiar participants. In contrast, most drivers in the unfamiliar group show positive loadings, while some exhibit negative loadings, indicating within-group variation in loading magnitude and opposite loading directions on the shared component under the adopted sign convention. Overall, this latent factor captures individual variation in loading magnitude and direction on the shared component, with route familiarity providing a useful *post-hoc* interpretation of the driver-mode pattern.

**Figure 7:**
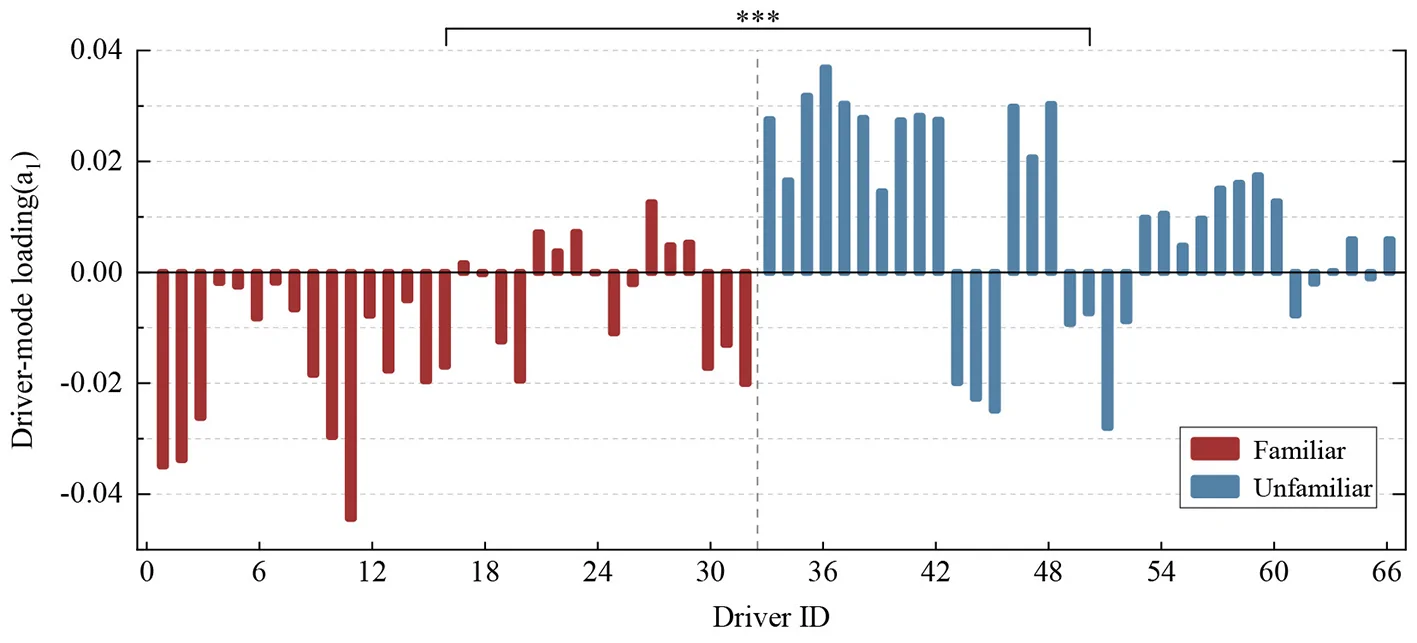
Individual driver-mode loadings on the rank-1 component (**a**_1_ vector). Exploratory comparisons between route-familiarity groups with Holm-adjusted *p*-value. ***p < 0.001.

### 4.2 Indicator contribution analysis

To reveal the structure of the primary factor along the indicator mode, this section examines the contribution weights of 13 multimodal driving indicators on the rank-1 component (**b**_1_ vector) obtained from the CP decomposition. This vector captures the dominant projection direction of the indicator dimension in the tensor structure. The magnitude of each loading reflects the contribution strength of a given indicator, while its sign indicates the directional alignment with the latent factor. Figure 8 presents the loading values sorted by absolute magnitude, highlighting the dominant components of the factor structure.

**Figure 8:**
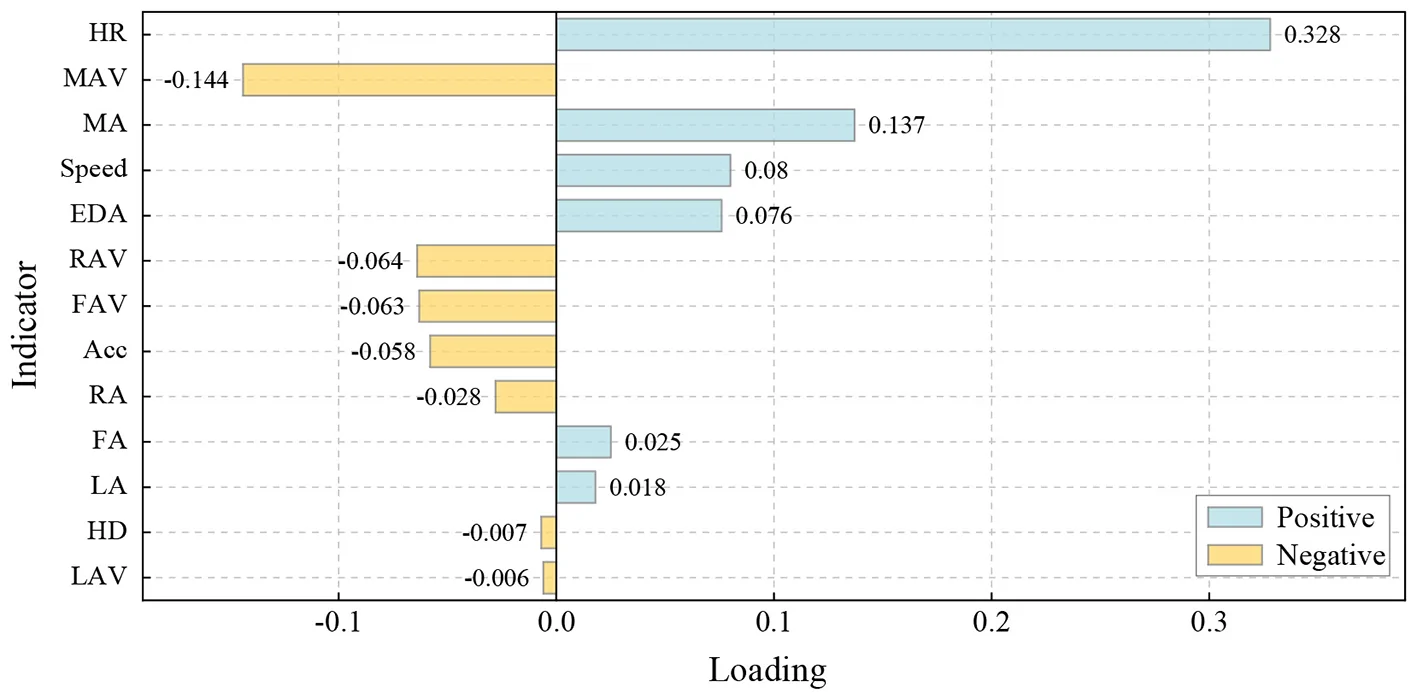
Indicator contribution on the rank-1 component (**b**_1_ vector).

Among all indicators, Heart Rate (HR) shows the highest positive contribution (0.328), suggesting that the latent factor is strongly associated with physiological arousal. AOI gaze-frame count in the main forward area (MA, 0.137), vehicle speed (Speed, 0.080), and electrodermal activity (EDA, 0.076) also contribute positively, indicating that the factor integrates both visual behavior and physiological response, possibly reflecting active demand regulation. In contrast, mean continuous gaze duration in the main forward area (MAV, –0.144) has the largest negative loading, indicating an opposite loading direction relative to MA. Similarly, mean continuous gaze duration in the right console region (RAV, –0.064) and mean continuous gaze duration in the dashboard region (FAV, –0.063) also exhibit negative loadings, indicating opposite loading directions within the component. Some indicators, such as headway distance (HD, –0.007) and mean continuous gaze duration in the left mirror region (LAV, –0.006), show limited contribution, suggesting minimal projection on this component. Overall, the dominant factor appears to integrate physiological and visual interaction features, primarily driven by HR and frontal gaze behavior, and coupled with behaviorally relevant indicators such as Speed and EDA, forming a workload-related psychological and behavioral response structure.

### 4.3 Spatial distribution analysis

Figure 9 illustrates the shared spatial profile of the selected rank-1 component across the 59 consecutive tunnel segments. High-response segments were operationally defined as segments whose spatial loadings fell within the upper 10% of the 59 segment loadings. Adjacent high-response segments were grouped as a high-response region, and the segment with the largest loading within each region was defined as the regional peak. Segments 17–18, 35, 52–53, and 58 were classified as high-response segments, forming four spatially separated high-response regions. The loading values range approximately from 0.0968 to 0.1526, showing no clear linear progression but instead exhibiting several localized peaks distributed across different spatial positions. Specifically, in the early stage (segments 1–20), the loading values show an initial rise, with a noticeable peak around segment 4, followed by a slight decline and another peak near segments 17–18. In the middle segment (segments 21–40), the curve maintains a relatively high fluctuation amplitude, with a prominent peak occurring at segment 35. The highest loading value 0.1526 is observed in this region, indicating it as one of the most concentrated high-response segments of the component. In the final segment (segments 41–59), the curve exhibits multiple peaks, with additional notable elevations around segments 52-53 and 58. Overall, the rank-1 component demonstrates localized structural responses across several spatial positions, suggesting the presence of meaningful spatial differentiation.

**Figure 9:**
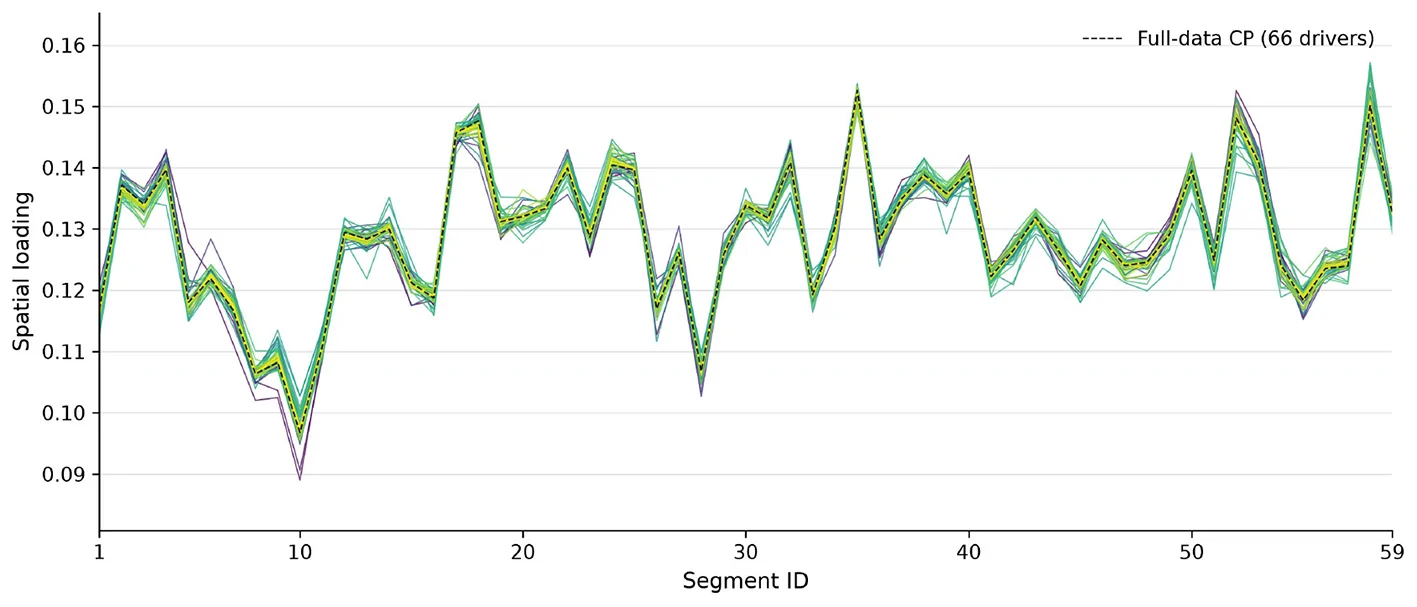
Spatial trend of the rank-1 component (**c**_1_ vector) and stability of the spatial loading under LODO analysis.

To assess the stability of the spatial loading, a leave-one-driver-out (LODO) sensitivity analysis was conducted. Each of the 66 drivers was omitted in turn, and the rank-1 CP model was refitted using the remaining 65 drivers under the same spatial segmentation. The LODO profiles were highly consistent across the 66 fits (as shown in Figure 9 colored lines), with Pearson correlation coefficients ranging from 0.9630 to 0.9999 and a mean of 0.9938. The results indicate that the overall spatial pattern was stable to the omission of individual drivers under the adopted segmentation scheme.

## 5 Discussion

This study examined latent multimodal response patterns in extra-long tunnels under label-free naturalistic driving conditions. The extracted dominant factor showed structured variation across the driver, indicator, and segment dimensions, suggesting that workload-related responses in extra-long tunnel driving vary across individuals, multimodal indicators, and spatial locations. The convergence of physiological activation, visual attention regulation, vehicle-control behavior, and spatial response patterns provides interpretive support for a workload-related reading of the factor. Therefore, the dominant factor provides a data-driven representation of workload-related multimodal responses in extra-long tunnel driving.

At the individual level, route familiarity was closely associated with the dominant latent response pattern. Under the adopted sign convention, male drivers unfamiliar with the tunnel showed higher driver-mode loadings on the shared component, suggesting that unfamiliar driving environments may impose additional perceptual and cognitive demands. This *post-hoc* differentiation by route familiarity strengthens the behavioral interpretability of the extracted factor, because unfamiliar routes are generally associated with greater demands for route prediction, risk monitoring, and attentional regulation. This finding is consistent with the view that uncertainty and increased information requirements in unfamiliar environments can intensify perceived workload. In contrast, familiar male drivers may have better expectations of tunnel geometry, lighting transitions, and potential conflict points, which may be associated with reduced mental effort for hazard anticipation and vehicle control. From a safety perspective, this result indicates that route familiarity is not only a background driver attribute, but also a factor that may influence individual differences in driver-mode loadings on the shared workload-related component in extra-long tunnels. Male drivers with limited familiarity may exhibit greater visual search, stronger attention allocation, and more frequent adjustment behaviors in response to the tunnel environment, making them a relevant group for future research on workload-related monitoring and driver-assistance applications.

The indicator-mode results further show that the dominant response structure was reflected through coupled physiological and visual responses. Heart rate and EDA contributed positively to the extracted factor, indicating that autonomic arousal was involved in the dominant latent response pattern. Together with the visual and behavioral indicators, these physiological responses suggest that the factor is associated with stress-related activation and regulatory effort under demanding driving conditions. This is consistent with previous findings that physiological activation is associated with increased task demand and mental workload. In addition, AOI gaze-frame count in the main forward area was positively associated with the factor, reflecting cumulative forward gaze allocation in the indicator-mode structure. Vehicle speed also contributed to the factor, indicating that variation in the latent response pattern was accompanied by behavioral regulation during tunnel driving. Several mean continuous gaze-duration indicators showed opposite loading directions from AOI gaze-frame-count indicators. This pattern suggests that workload-related visual behavior may involve different gaze strategies, including changes in cumulative forward gaze allocation and mean continuous gaze duration across visual regions. Under demanding driving conditions, male drivers may redistribute visual attention to gather sufficient information for decision-making and maintain operational stability. Therefore, the indicator-mode structure supports interpreting the dominant component as a workload-related multimodal response pattern integrating physiological, behavioral, and visual measures.

The spatial-mode results indicate that the spatial loadings of the dominant latent component vary along the driving route. Elevated response loadings were observed around the entrance segments, the exit segments, and a central interior region. The entrance and exit elevations are consistent with prior findings that tunnel transition zones often increase cognitive and physiological demands because of abrupt luminance changes, black-hole and white-hole effects, and rapid changes in visual adaptation. These transition zones are often associated with rapid adjustments in visual attention, speed control, and risk perception, which may contribute to segment-level increases in multimodal response. More importantly, elevated-response segments were also identified within the tunnel, suggesting that workload-related responses may emerge across multiple spatial locations. In prolonged enclosed environments, visual monotony, restricted spatial cues, acoustic effects, curvature changes, or local lighting variations may contribute to accumulated perceptual demand and changes in the observed response pattern. Segment-level response mapping may provide exploratory information about locations associated with increased perceptual demand or potential driver-support needs and identify candidate high-response segments that may warrant further safety investigation. Because physiological responses, particularly EDA, may lag behind the triggering stimuli, the approximately 100-meter segment averages indicate where responses were recorded and do not necessarily identify the precise locations of the stimuli that elicited them.

These results also connect the present study to affective processes in transport safety. The positive contributions of HR and EDA suggest that autonomic activation may be involved in the extracted latent pattern, while the accompanying visual-attention and vehicle-control indicators suggest possible attention regulation and behavioral adaptation during tunnel driving. Together, these multimodal associations are consistent with an arousal-related interpretation, in which physiological activation may support vigilance and regulatory effort under demanding conditions. In extra-long tunnels, abrupt illumination changes, spatial enclosure, restricted visual references, and prolonged monotony may increase perceived environmental uncertainty and monitoring demand, which could contribute to stress-related arousal and adaptive regulation. Taken together, these multimodal associations suggest that the dominant component may reflect an affect-relevant and workload-related response pattern involving physiological activation, attention regulation, and vehicle-control behavior during extra-long tunnel driving.

From a practical perspective, these findings may provide useful insights for both driver-level and segment-level safety management in extra-long tunnels. Future studies may evaluate enhanced route guidance, clearer advance information, or in-vehicle workload-related support for male drivers with stronger responses, especially those unfamiliar with the tunnel environment. Tunnel segments with elevated response loadings may be treated as candidate high-response segments that warrant further safety investigation to inform future consideration of lighting adjustment, visual reference enhancement, speed guidance, warning placement, and other targeted measures. Compared with approaches that rely on an overall workload score, the proposed multi-way analysis provides three complementary views of the latent response structure: variation in driver-mode loadings on the shared component, indicator contributions to that component, and its shared spatial distribution along the tunnel. This may support a more targeted understanding of workload-related psychological responses across drivers and tunnel segments.

## 6 Conclusion and future work

This study examined latent multimodal response patterns in extra-long tunnels from a label-free, multi-way analysis perspective. These patterns were interpreted as affect-relevant and workload-related based on convergent evidence. Multimodal naturalistic driving data, including physiological responses, driving behavior, and gaze measures, were organized as a third-order tensor to represent the interaction among individual drivers, indicators, and spatially segmented tunnel sections. Through tensor-based latent factor extraction, the study characterized response patterns that are difficult to capture using single indicators, aggregated workload scores, or label-dependent modeling approaches. The dominant component showed structured variation across male drivers, indicators, and spatial modes. Driver-mode loadings showed differences between familiar and unfamiliar male drivers. Physiological arousal and visual-attention measures, particularly HR, EDA, and frontal gaze-frame and mean-duration indicators, contributed strongly to the extracted pattern. Higher component loadings appeared not only near tunnel entrance and exit areas but also in several interior sections. These findings suggest that the multimodal response pattern interpreted as affect-relevant and workload-related may vary across both transition and interior tunnel zones.

Overall, this study provides an interpretable and exploratory approach for characterizing latent multimodal response patterns and their driver- and segment-level variations in extra-long tunnels under naturalistic driving conditions. The physiological, visual, behavioral, spatial, and driver-level patterns support an affect-relevant and workload-related reading of the dominant component. The findings may provide exploratory evidence for future assessment of warning placement, lighting improvement, speed guidance, visual reference design, and driver-state monitoring in tunnel safety management.

Several limitations should also be acknowledged. The data were collected from a single tunnel route, and all drivers were male, which may limit the generalizability of the findings to other tunnel types, traffic conditions, and driver populations. Future studies should include female drivers to validate the identified response patterns. In addition, richer environmental variables, such as lighting conditions, curvature, sight distance, and traffic density, were not explicitly incorporated into the current analysis. The present framework was also based on offline data analysis and did not model temporal dynamics or causal relationships. Future research should validate the identified workload patterns across different tunnel environments, integrate road and traffic characteristics into the modeling framework, and explore dynamic or online extensions for real-time workload monitoring and safety intervention.

Because the selected model is rank 1, individual differences are represented through driver-mode loadings on shared indicator and spatial profiles rather than through independently estimated driver-specific profiles. Individual structures beyond the dominant shared component may therefore remain unrepresented.

## Data Availability

The raw data supporting the conclusions of this article will be made available by the authors, without undue reservation.
